# Transposable Elements Are a Significant Contributor to Tandem Repeats in the Human Genome

**DOI:** 10.1155/2012/947089

**Published:** 2012-06-24

**Authors:** Musaddeque Ahmed, Ping Liang

**Affiliations:** Department of Biological Sciences, Brock University, St. Catharines, ON, Canada L2S 3A1

## Abstract

Sequence repeats are an important phenomenon in the human genome, playing important roles in genomic alteration often with phenotypic consequences. The two major types of repeat elements in the human genome are tandem repeats (TRs) including microsatellites, minisatellites, and satellites and transposable elements (TEs). So far, very little has been known about the relationship between these two types of repeats. In this study, we identified TRs that are derived from TEs either based on sequence similarity or overlapping genomic positions. We then analyzed the distribution of these TRs among TE families/subfamilies. Our study shows that at least 7,276 TRs or 23% of all minisatellites/satellites is derived from TEs, contributing *∼*0.32% of the human genome. TRs seem to be generated more likely from younger/more active TEs, and once initiated they are expanded with time via local duplication of the repeat units. The currently postulated mechanisms for origin of TRs can explain only 6% of all TE-derived TRs, indicating the presence of one or more yet to be identified mechanisms for the initiation of such repeats. Our result suggests that TEs are contributing to genome expansion and alteration not only by transposition but also by generating tandem repeats.

## 1. Introduction

Over half of the human genome consists of repeat elements. The two types of repeat elements that are prevalent in human genome are tandem repeats (TRs) of sequences ranging from a single base to mega bases and interspersed repeats that mainly include transposable elements (TEs). The tandem repeats are classified in three major classes based on the size of the repeated sequence: microsatellites for short repeat units (usually <10 bp), minisatellites for head-to-tail tandem repeat of longer units (>10 and <100 bp), and satellites for even larger units (>100 bp). Among all types of tandem repeats, minisatellites and microsatellites have gained increasing attention over the past decade due to their contribution to intraspecies genetic diversity and use as genetic markers in population genetic studies. These repeat sequences are widespread in all eukaryotic genomes (reviewed in [1]) from yeast to mammals and often are highly polymorphic in populations of the same species. Consequently they are often used as a marker in numerous genotypic tests, for example, in forensic fingerprinting [2–5], in population genetics [6], and in monitoring of DNA damage induced by ionizing radiation [7]. Minisatellites lately have been of particular interest because their expansion has been implicated in alteration of gene expression often leading to diseases [8]. Origin and expansion of microsatellites have been well studied and the most widely accepted mechanism underlying microsatellites states that the initiation takes place by chance, and then they are expanded by slipped-strand mispairing [9]. On the other hand, origin of minisatellites and satellites is very difficult to study, and even though a significant progress has been made in understanding the expansion and contraction of such repeats, a number of major aspects are still unresolved (reviewed in [10]). For expansion and contraction of longer repeats, several lines of evidence suggest gene conversion during meiosis as the major mutational force rather than replication slippage [11, 12]. As for the direction of expansion, it has been found to be usually polar, that is, addition of new repeat unit occurs only at one end [13].

While the expansion of longer sequences is well studied, the origin or initiation of such repeats is difficult to understand because it is very unlikely for duplication of such long repeats to initiate by chance. There are two models that attempt to explain the initiation of minisatellites/satellites. One model postulates slipped-strand mispairing at noncontiguous repeats when there is a pause during replication [14]. A key feature of this model is that expanded TR's terminal repeat unit should be “incomplete”, that is, shorter than other repeat units by a number of nucleotides. The second model postulates that when a long sequence is flanked by direct repeats of 5–10 bp, it can be duplicated by replication slippage or unequal crossing-over [15].

The other major class of repeats in the genome, transposable elements, are ubiquitous in both prokaryotes and eukaryotes. TEs can mutate genomes by transposing to new locations or by facilitating homology-based recombination due to their abundance in the genome. At least 44% of the entire human genome is composed of TEs that belong to at least 848 families or subfamilies (reviewed in [16]). Majority of the TEs in humans is contributed by two classes, L1 and Alu. When human genome was compared with chimpanzee genome, more than 10,000 species-specific insertions were identified, over 95% of which is contributed by L1, Alu, or SVA [17–20]. SVA is a composite element that is derived from three other repeat elements: SINE-R, VNTR, and Alu. A small number of human-specific TE insertions are also contributed by Human Endogenous Retrovirus-K (HERV-K) [18]. These human-specific TE insertions indicate that these TE families are/were active after the divergence of humans from chimps ~6 million years ago. Alu family has three large subfamilies, AluJ, AluS, and AluY, with their ages being considered very old, old, and young, respectively.

Even though the effects of TRs and TEs are well studied and understood individually, there have not been many studies that investigated the relationship between these two classes of repeat sequences. To our knowledge, the first study linking tandem repeats and transposable elements was reported by Jurka and Gentles [21] in an attempt to identify the origin and diversification of minisatellites derived from Alu sequences. Their work demonstrates how Alu sequences can be tandemly repeated because of short direct repeats flanking the repeat arrays. Later Ames et al. [22] also reported 111,847 TRs overlapping with interspersed repeat sequences in an attempt to compare between single-locus TRs and multilocus TRs. They included microsatellites and all types of interspersed repeats but did not analyze the relationship between TRs and TEs any further. In the current study, we for the first time assessed the genome-wide contribution of TEs to the generation of minisatellites/satellites TRs, revealing that at least 7,276 TRs or 23% of all minisatellites/satellites was derived from TEs. We compared and identified the classes of TEs that are more prone for generating TRs, and we also examined the mechanisms for initiation and expansion of the tandem repetition of the TEs.

## 2. Materials and Methods

### 2.1. Collection of TR and TE Data in the Human Genome

The Tandem Repeat data was downloaded to our local server from the Tandem Repeat Database (TRDB) (http://tandem.bu.edu/cgi-bin/trdb/trdb.exe) that documents the genomic positions of each repeat, consensus repeat sequence, and number of repeats among an array of useful information [23]. The consensus sequences of all families and subfamilies of TEs were downloaded from RepBase (http://www.girinst.org/repbase/) [24]. The positions of all individual TEs in the human genome were downloaded from UCSC Genome Annotation Database for genome version hg19 (http://hgdownload.cse.ucsc.edu). The UCSC hg19 (NCBI Build 37) version of human genome sequence was downloaded from UCSC website and was compiled to create a database for BLAST. Algorithms to perform all analytic tasks were developed in-house using the programming language Perl on Unix platform. 

### 2.2. Identification of TE-Derived TRs

Output from TRDB for all TRs in the human genome was filtered using an in-house Perl script such that they meet the following criteria: repeat unit length ≥20 bp, GC content ≥40%, repeat number ≥2, and sequence similarity among the repeat units in an array ≥95%. Many satellites are parts of a larger satellites which cause redundancy in the final set; to avoid this, overlapping TR arrays are separated and the TRs with smallest period from each set of overlapping arrays were used for the subsequent analyses. A TR is considered to be derived from a TE if it meets one of the following two criteria: (1) the TR repeat unit sequences have a minimum of 70% similarity with the consensus sequence of a human TE; (2) a TR locus overlaps in position with a TE by at least one period. To identify TRs that are at least 70% similar to a TE, the targeted TR repeat sequences were aligned against the TE consensus database using BLAST by setting e-value at 10^−6^, mismatch penalty at −1 and word size at 7. In the second method of identification, the starting and ending genomic positions of a tandem repeat arrays were cross-checked using an in-house PERL script. Any TR overlapping a TE by the length of at least one TR period was considered TE derived. Clustering all selected TRs was performed by using the NCBI BlastClust tool with a maximal sequence length disparity of 10% and a minimal sequence similarity of 85% among the members of a cluster.

### 2.3. Identification and Distribution of TE Families Contributing to TR

The TR repeat unit was aligned pairwise with its corresponding candidate parent TE using the NCBI bl2seq tool with zero penalty for alignment gap to identify the region of the TE that is duplicated. The contribution of each TE family and subfamily to TR is evaluated not only by the total number of TRs contributed but also based on the relative TE abundance, which is represented as the percentage of TE in the subfamily that are contributing to TR. This relative number is calculated by dividing the actual number of TE loci involving TR with the total loci of that TE and multiplying by 100.

### 2.4. Identification of Sequence Similarity among Repeat Units and with Orthologous Sequences in Other Primate Genomes

To identify the possible mechanism of TR expansion, 5 AluJ-derived TRs with more than 15 repeat units were randomly chosen for manual analysis. Each individual repeat unit was aligned to hg19 using BLAT with default parameters to identify all genomic regions that it matches with. All aligned regions were sorted according to the similarity score to identify the best match. If the expansion occurred due to sequential duplication of the repeat unit, the best matching region would be the repeat unit adjacent to the test sequence. If a TR was generated along with retrotransposition, that is, simply representing a copy of a TR in the parent TE somewhere else, then we would expect to see better sequence similarity elsewhere in the genome than among repeats in the same array. The tandem arrays were then aligned with the latest version of chimpanzee, orangutan, gorilla, and marmoset genome sequences using UCSC genome browser in an attempt to find similar repeat arrays in other primates. If the expansion occurred slowly through evolution, each repeat array was expected to have partial to no match with other primate genomes. Moreover, TRs with higher number of repeat units were expected to had accumulated more mutations than TRs with smaller number of repeat units due to their residence in the genome for a longer time. To test whether TRs with a larger number of repeats are older than the TRs with a small number of repeats, we surveyed the maximum sequence divergence among the repeat units in TRs. To do this, we classified all non-LTR12 and non-L1PA TE-derived TRs in two classes: one with ≤3 units and the other with ≥10 units. Repeat units in each TR were then separated using Perl script and aligned pairwise to one another to create an evolutionary distance matrix among the repeat units using CLUSTALW (downloaded for Linux platform from ftp://ftp.ebi.ac.uk/pub/software/clustalw2) [25]. The distance is calculated by dividing the total number of mismatches between two units with total number of matched pairs. The maximum divergence for each TR was obtained from its corresponding distance matrix.

## 3. Results and Discussion

In this study, we seek to perform a genome-wide survey of the contribution of transposable elements to the generation of tandem repeats and examine the possible mechanisms. The starting point of this study consisted of the output data from the Tandem Repeats Database which provides a compilation of all tandem repeats in human genome ranging from 1 bp to 2000 bp in size of the repeat unit. For the latest assembly of human reference genome (NCBI build 37 or Hg19), TRDB annotates 31,472 minisatellites and satellites (both will be called minisatellites hereafter for simplicity) with repeat unit length more than 20 bp, minimum GC content of 40%, and minimal number of repeats of 2 and has at least 95% identity among the repeat units in an array. A minimal 40% of GC content was applied to eliminate TRs that contain mainly low complexity or simple repeat sequences, which can derive from poly (dT) or poly (dA), present frequently in non-LTR retrotransposable elements as the 3′-end polyA track or the internal sequence of Alu or SVA. Of the 31,472 minisatellites, 7,276 (23.12%) were detected as being derived from transposable elements either by sequence similarity with TE consensus sequences or by overlapping an annotated genomic TE region by at least one period (The complete TR list is provided in Supplementary Table 3 Supplementary mareial avaliable online at doi:10.1155/2012/947089). The TE-derived minisatellites were then classified into 5,932 clusters based on their sequence similarity, with each cluster representing tandem repeats that are likely to have been derived from or related to a particular TE. Among the 5,932 clusters, 185 contain similar sets of tandem repeats that are found in more than one locus in the whole genome and thus are termed as multilocus TRs or “mlTRs” following the nomenclature proposed by Ames et al. [22], and 5,747 clusters contain TE-derived TRs that are present only in one locus in the genome and thus are termed as single-locus TRs or “slTRs”. These 7,276 TE-derived TRs contribute to a total of 1.05 Mb of sequence or ~0.32% of the human genome, and we believe that these numbers represent a underestimate of such events that have happened in the human genome, since we may fail to detect a lot of old TRs as a result of high sequence divergence (see more discussion later). 

### 3.1. Younger and More Active TEs Are More Susceptible for Tandem Duplication

**Figure 1:**
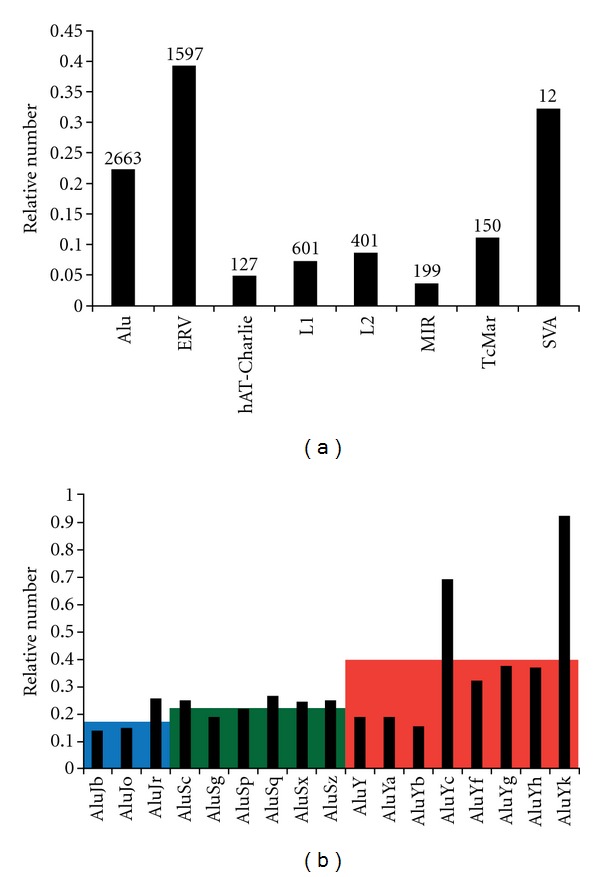
Relative abundance of major families and subfamilies of TEs that generate TRs. Relative abundance is calculated by dividing the number of TE-derived minisatellites by the total number of members in that TE family. (a) Relative abundance of major families of TR-associated TEs. The actual number of TE-derived TRs is at the top of each bar. (b) Relative abundance of subfamilies of TR-associated Alus. The color-shaded boxes are average relative abundance for the group with blue for AluJ, green for AluS, and orange for AluY. It is evident that the average relative abundance increases from AluJ to AluS to AluY.

Almost 19% of the TE-TRs (1,374 of 7,276) is derived from LTR12 and L1PA subfamilies of retrotransposons. This was expected due to the internal tandem repeat in the consensus sequence of these two subfamilies. To avoid bias in assessing the general trend, we treated these separately from those associated with other TE subfamilies. For the other TEs, the most number of TRs (2663) were found to be derived from Alu, while ERVs and L1 had 1597-and 601-associated TRs, respectively. Since the abundance for each TE subfamily is different in the human genome, the number of TEs for each subfamily of TEs was normalized for the total number of TEs in that subfamily in the genome. After normalization, Human Endogenous Retroviruses (HERVs), including the internal viral sequences and LTRs, exhibit a relatively higher percentage of tandem duplication (39%), with almost 90% of members belonging to HERV-K subfamily, which is the youngest and most active ERV. Even though the actual number of SVA-derived TRs is as small as 12, when normalized, SVA has the second highest relative abundance (32%) in terms of generating TRs. Following HERV and SVAs, Alus are the TE classes with the third most abundant tandem repeats, and all of them belong to the younger and more active classes of TE in the human genome (Figure 1(a)). When the subfamilies of Alu are examined for relative abundance of tandem repeats, all subfamilies exhibit somewhat similar abundance, with AluY seeming to show slightly higher abundance (Figure 1(b)). However, the mean abundance of the three major subfamilies of Alu: AluJ, AluS and AluY shows a clear increment of relative TR abundance from AluJ (0.18) to the intermediate AluS (0.24) to AluY (0.40). This also follows the trend of younger/more active TEs generating a higher number of TRs as AluJ is the oldest subfamily of Alus, while AluY is the youngest and most active subfamily of Alus. The age of AluJ has been dated back to 26 million years ago [26] and no species-specific AluJ activity has been identified in the comparative studies between humans and chimpanzees. AluS diverged from AluJ later and only 262 new AluS insertions have been identified in humans that happened within last 6 million years ago, which is a fraction of the total AluS insertions annotated in the human genome [18]. The youngest family of Alus is AluY, and they are believed to be the most active Alu family in the present human genome. The trend of increasing relative TR abundance from older subfamilies to newer subfamilies of TEs may indicate that the initiation of TE-derived TRs, at least for a large number of cases, can potentially be associated with the retrotransposition process of TEs. In other words, the positive association between abundance of TE-derived TRs and transposition activity level of TEs may suggest that retrotransposition contributes to the initiation of TRs, despite the possibility that the lower relative abundance of TRs on older TEs could also be due to recombination-mediated deletion and/or lower detection because of sequence divergence. 

### 3.2. Older TEs Have a Larger Number of Repeat Units Than Younger Ones

**Figure 2:**
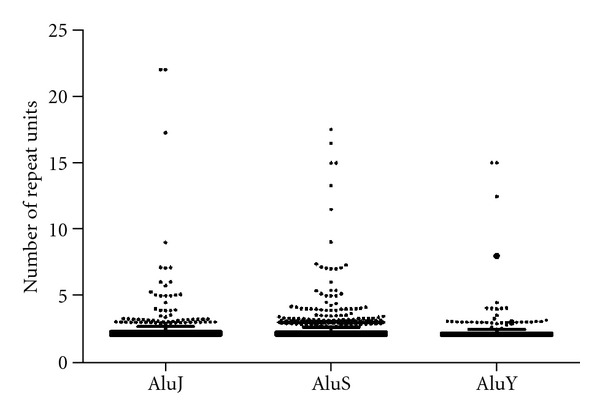
Box and Whiskers plot of the number of repeats for TRs derived from the three major classes of Alu. The average number of repeat units decreases from AluJ (2.42) to AluS (2.31) to AluY (2.30).

The initiation of TR expansion occurs more often with younger classes of TEs (Figure 1). However, once a region is repeated at least once, the increase in the number of the repeat may occur by previously reported mechanisms for such events (further discussed later in the section). When the number of repeats for each major subclass of Alu is plotted in a graph, a steady decrease in number of repeats from older to newer class of Alus becomes, clear (Figure 2). The AluJ has a mean number of repeat units of 2.42, AluS has 2.31, and AluY has 2.30. The differences in variance among these classes of Alus were found to be statistically significant (*P* < 0.0001) when tested using the statistical method of Analysis of Variance (ANOVA). However, the difference in mean number of repeat units between AluS and AluY is not statistically significant in a two-tailed *t*-test. But this can be largely due to the fact that the total number of TRs generated by AluS is more than four times higher than that by AluY with majority having a repeat number below 3. Furthermore, the evolutionary distance between AluS and AluY is less than that between AluJ and AluS [27]. When older AluS subfamilies (AluSx, AluSg, AluSp and AluSq) were examined, 8.11% of their associated TRs has more than 3 repeat units, while only 6.70% of TRs from AluY has more than 3 repeat units (data not shown) and the newest AluY elements: AluYa and AluYb have no TRs with more than 3 repeat units. This decrease in repeat number from older to younger families of TEs can be explained as the expansion of repeat units is a slow process, and it takes longer time to generate more TR repeats. When the TE-derived TRs with a larger number of repeats were aligned against the orthologous sequences from other primates, only a portion of the total repeat is found in the outgroups. In Supplementary Figure 1, a 17 tandem repeats of 52 bp from AluJo (from 226 to 278 bp of the consensus sequence) are aligned against the corresponding sequences in the outgroup genomes, and only a portion of the total TR is matched in the these genomes. Since AluJo appeared in primates 26 million years ago [26], the extra repeat units can be explained as further extension of the common repeat units in the human genome after the diversion from chimps by *in situ* duplication rather than by transposition. This is further supported by our observation in examining 5 randomly chosen Alu-derived TRs with a minimal number of repeat units of 15, in which the repeat units in an array of TR are best aligned against each other than any other region in the genome, indicating that one unit was used as the source of the other for duplication in a local manner. When the mlTRs were investigated, 45 out of 185 mlTRs were found to be variable in number of tandem repeat units in different loci. With exception of one, all of these mlTR clusters follow the same trend of decreasing number of loci with increase in the number of repeat units (Supplementary Table 1). This again indicates that the expansion of repeat units of a TR may occur sequentially with time, for which in a cluster of mlTRs, the TRs with higher number of repeat units are seen in lesser number of loci. When LTR12-derived TRs are analyzed, the number of repeats in the internal sequence is found to be variable throughout the genome. Complying with the relationship seen between number of repeats and number of occurrence in non-LTR12 mlTRs, the larger the number of repeated sequences, the less the number of loci. This provides evidence that these duplication events have taken place throughout the evolution and the repeats are possibly increased sequentially in number. Also for this reason, an entire TR generated by the older TEs or part of a TR that has existed for much longer time have been subject to more mutations/deletions than the younger ones. In other words, the TRs with more repeat units should accumulate more mutations than TRs with smaller number of repeat units because of their longer residence in the genome. When the evolutionary distance among repeat units in TRs with ≤3 repeat units and ≥10 repeat units was examined, the mean highest distance found in TRs with ≤3 units was 0.5330 while that of TRs with ≥10 units was 0.8049 (Supplementary Figure 2). The difference in maximum divergences among repeat units between the short and long TRs is statistically significant (two tailed *t*-test *P* < 0.0001). This provides direct evidence that TE-derived TRs are expanded gradually throughout evolution. Some of these TRs or TR repeats may have been mutated to a point where they have become undetectable as tandem repeats by the current algorithms. For this reason, the number and/or the length of TRs derived from TEs may have been underestimated.

### 3.3. Certain TE Regions Can Act as Hotspots for Tandem Duplication

**Figure 3:**
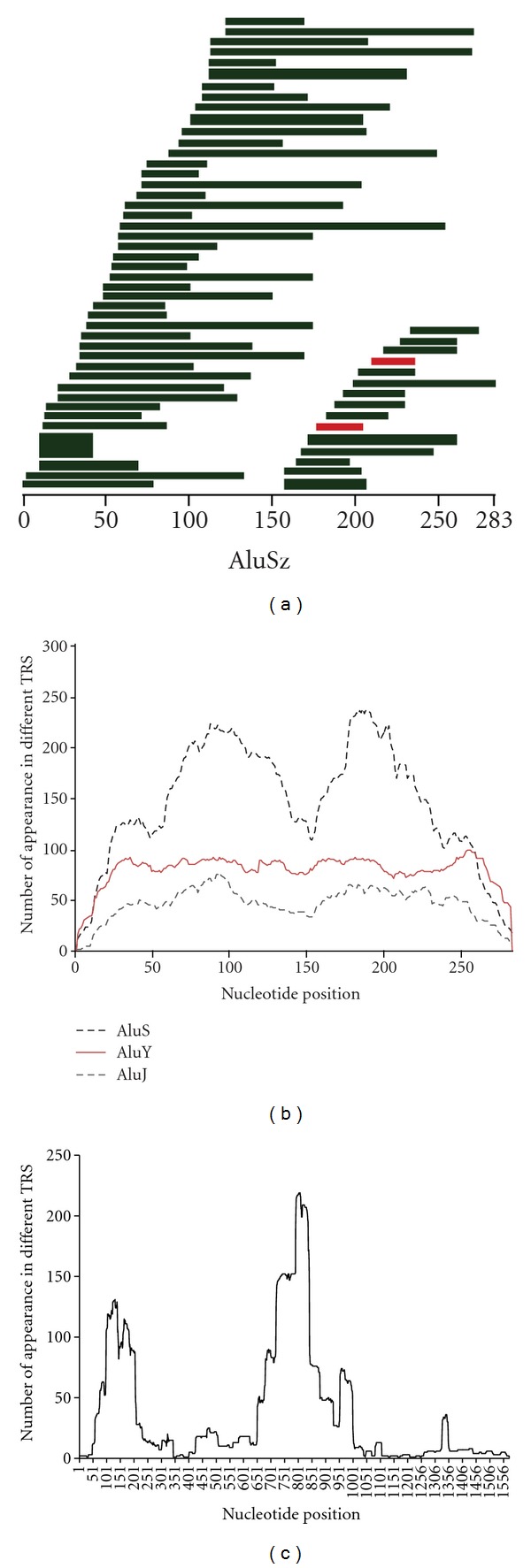
Regions of TE that are involved in generating TRs for Alus and LTR12. (a) Representation of a selected number of fragments of AluSz that have generated TRs. Selection was made randomly to demonstrate that the repeat can occur from any region of a TE. The height of each bar is proportional to the number of repeats. Green colored regions are duplicated in 2 loci, and red colored regions are duplicated in 3 loci; (b) The number of TRs spanning each nucleotide of AluS, AluJ, and AluY; (c) The number of TRs spanning each nucleotide of LTR12.

To see whether hotspots of TRs exist in the genome or in specific region of TEs, we plotted the TE-derived TRs in the whole genome, and no obvious hotspots were seen in the genome (Supplementary Figure 3). When the positions of the repeated regions are plotted in AluJ and AluY, no TR hotspot was identified (Figures 3(a) and 3(b)). But there are two regions (59 to 137 bp and 176 to 206 bp) found in the AluS consensus sequence that are spanned by comparatively more TRs than other regions (Figure 3(b)). There are also two distinct hotspots observed for LTR12 from 99 to 182 bp and from 719 to 841 bp (Figure 3(c)). This may be due to the fact that TR existed in the original LTR12 sequences and the TRs were propagated also by transposition, different from other TE-derived TRs where initiation and expansion occurred at or after individual TE insertion.

### 3.4. Multiple Mechanisms for Generation of TE-Derived TRs

Of the 7,276 TE-derived TRs, 159 TRs have incomplete terminal repeat unit that is smaller in size than the other unit(s) by maximum of 10%, that is, if the unit length of the TR is 100 bp, the terminal unit's length is between 90 to 99 bp. Initiation of these TRs can follow the mechanism of slipped-strand mispairing proposed by [14], as having an incomplete or truncated repeat unit at the end of the repeat array is a key feature of this mechanism. Among other TE-derived TRs, 300 were found to have flanked by direct repeats of size 5–20 bp. The initiation of such TRs can be explained by the mechanism proposed by Haber and Louis [15]. According to that model, replication slippage including gene conversion or unequal crossing over during meiotic replication can cause gain or loss of a copy of the region flanked by such small direct repeats. The majority of these flanking repeats is of size at 7 bp, which is consistent with this model (Supplementary Table 2) [21, 28]. These two established mechanisms may explain initiation of only 6% of all TE-derived TRs. The rest 6,817 TRs are not flanked by direct repeats or incomplete terminal repeat, with the majority have only two repeat units. Thus these 6,817 TRs are unaccountable by the currently established mechanisms and hence are likely subjected to one or more yet to be identified mechanism(s). Among these, 136 TRs exhibit a specific pattern of repeat of a partial Alu (average length of 88.6 bp) adjacent to a full or near full length Alu (at least 300 bp). The duplication of the partial Alu sequence at the 5′ end of a TE may occur due to recombination or unequal crossing-over due to the presence of an endonucleolytic site immediately adjacent to the 5′ end of the TE. This endonucleolytic site is the target of LINE-1 endonuclease and can function as recombination hotspots [29]. It has also been proposed that when the endonuclease acts on such targets, single-strand nicks can be generated in DNA to promote recombination [30]. In addition to such well-defined preintegration endonuclease target sequences, potentially kinkable dinucleotides such as TA, CA, and TG can also promote nicking, consequently promoting recombination [31, 32], and thus may serve as potential mechanism of TR initiation.

## 4. Concluding Remarks

While transposable elements are known for genomic rearrangement and expansion of the genome by transposition, we show in this study that they also play a role in genome expansion and alternation by contributing to tandem repeats. Over 20% of all minisatellites/satellites is contributed by TEs, constituting a total length of 1.05 million base pairs in the human genome, and according to the results of this study, this number is and will be increasing. 

Results from this study suggest that the tandem repetition of full or partial TEs can be triggered during retrotransposition, and once it is duplicated, the expansion of the repeat units can slowly occur through time. While a small portion (6%) of TE-derived TRs can be explained by one of the mechanisms postulated so far, the mechanism(s) for the majority is yet to be identified, thus our results present the need for identifying new mechanisms underlying the TE-derived TRs initiation and expansion. Furthermore, no study has yet revealed the detailed nature of the recombination hotspots adjacent to the minisatellites in terms of their DNA primary structure, plasticity or secondary structure, and thermal stability or functionality [11]. Understanding these phenomena will definitely help identifying exact mechanism(s) of tandem repeats derived from transposable elements.

## Supplementary Material

Supplementary Material 1: Containing supplementary figures 1–3 and tables 1-2.
Supplementary Figure 1: A schematic comparison for 17-repeat TR array involving the 226–278 bp region in a Alujo among difference species.
Supplementary Figure 2: Box and Whiskers plot of maximum divergence among repeat units in TRs with ≤3 and ≥10 repeat units.
Supplementary Figure 3: Genomic locations of all TE-derived TRs.
Supplementary Table 1: The number of mlTRs at different repeat units for mlTR clusters.
Supplementary Table 2: The distribution of direct repeat length for TE-derived TRs with identifiable direct repeats.
Supplementary Material 2: Contains Supplementary table 3 which lists all 7,276 TE-derived TRs in a Microsoft Excel sheet.

## References

[1] Charlesworth B (1994). Genetic recombination: patterns in the genome. *Current Biology*.

[2] Jeffreys AJ, Wilson V, Thein SL (1985). Individual-specific “fingerprints” of human DNA. *Nature*.

[3] Tamaki K, Huang XL, Yamamoto T, Uchihi R, Nozawa H, Katsumata Y (1995). Applications of minisatellite variant repeat (MVR) mapping for maternal identification from remains of an infant and placenta. *Journal of Forensic Sciences*.

[4] Spurr NK, Bryant SP, Attwood J (1994). European Gene Mapping Project (EUROGEM): genetic maps based on the CEPH reference families. *European Journal of Human Genetics*.

[5] Jeffreys AJ, Pena SD (1993). Brief introduction to human DNA fingerprinting. *Experientia*.

[6] Armour JAL, Anttinen T, May CA (1996). Minisatellite diversity supports a recent African origin for modern humans. *Nature Genetics*.

[7] Bois P, Jeffreys AJ (1999). Minisatellite instability and germline mutation. *Cellular and Molecular Life Sciences*.

[8] Sutherland GR, Baker E, Richards RI (1998). Fragile sites still breaking. *Trends in Genetics*.

[9] Levinson G, Gutman GA (1987). Slipped-strand mispairing: a major mechanism for DNA sequence evolution. *Molecular Biology and Evolution*.

[10] Bois PRJ (2003). Hypermutable minisatellites, a human affair?. *Genomics*.

[11] Murray J, Buard J, Neil DL (1999). Comparative sequence analysis of human minisatellites showing meiotic repeat instability. *Genome Research*.

[12] Richard GF, Pâques F (2000). Mini- and microsatellite expansions: the recombination connection. *EMBO Reports*.

[13] Jeffreys AJ, Tamaki K, MacLeod A, Monckton DG, Neil DL, Armour JAL (1994). Complex gene conversion events in germline mutation at human minisatellites. *Nature Genetics*.

[14] Taylor JS, Breden F (2000). Slipped-strand mispairing at noncontiguous repeats in Poecilia reticulata: a model for minisatellite birth. *Genetics*.

[15] Haber JE, Louis EJ (1998). Minisatellite origins in yeast and humans. *Genomics*.

[16] Mills RE, Bennett EA, Iskow RC, Devine SE (2007). Which transposable elements are active in the human genome?. *Trends in Genetics*.

[17] Hedges DJ, Callinan PA, Cordaux R, Xing J, Barnes E, Batzer MA (2004). Differential Alu mobilization and polymorphism among the human and chimpanzee lineages. *Genome Research*.

[18] Mills RE, Bennett EA, Iskow RC (2006). Recently mobilized transposons in the human and chimpanzee genomes. *American Journal of Human Genetics*.

[19] Watanabe H, Fujiyama A, Hattori M, Taylor T, Toyoda A, Kuroki Y (2004). DNA sequence and comparative analysis of chimpanzee chromosome 22. *Nature*.

[20] Wang J, Song L, Gonder MK (2006). Whole genome computational comparative genomics: a fruitful approach for ascertaining Alu insertion polymorphisms. *Gene*.

[21] Jurka J, Gentles AJ (2006). Origin and diversification of minisatellites derived from human Alu sequences. *Gene*.

[22] Ames D, Murphy N, Helentjaris T, Sun N, Chandler V (2008). Comparative analyses of human single- and multilocus tandem repeats. *Genetics*.

[23] Gelfand Y, Rodriguez A, Benson G (2007). TRDB—the tandem repeats database. *Nucleic Acids Research*.

[24] Jurka J, Kapitonov VV, Pavlicek A, Klonowski P, Kohany O, Walichiewicz J (2005). Repbase Update, a database of eukaryotic repetitive elements. *Cytogenetic and Genome Research*.

[25] Chenna R, Sugawara H, Koike T (2003). Multiple sequence alignment with the Clustal series of programs. *Nucleic Acids Research*.

[26] Kapitonov V, Jurka J (1996). The age of Alu subfamilies. *Journal of Molecular Evolution*.

[27] Churakov G, Grundmann N, Kuritzin A, Brosius J, Makaowski W, Schmitz J (2010). A novel web-based TinT application and the chronology of the Primate Alu retroposon activity. *BMC Evolutionary Biology*.

[28] Nishizawa S, Kubo T, Mikami T (2000). Variable number of tandem repeat loci in the mitochondrial genomes of beets. *Current Genetics*.

[29] Babcock M, Pavlicek A, Spiteri E (2003). Shuffling of genes within low-copy repeats on 22q11 (LCR22) by Alu-mediated recombination events during evolution. *Genome Research*.

[30] Gentles AJ, Kohany O, Jurka J (2005). Evolutionary diversity and potential recombinogenic role of integration targets of non-LTR retrotransposons. *Molecular Biology and Evolution*.

[31] Jurka J, Klonowski P, Trifonov EN (1998). Mammalian retroposons integrate at kinkable DNA sites. *Journal of Biomolecular Structure and Dynamics*.

[32] Mashkova TD, Oparina NY, Lacroix MH (2001). Structural rearrangements and insertions of dispersed elements in pericentromeric alpha satellites occur preferably at kinkable DNA sites. *Journal of Molecular Biology*.

