# Supplementary material for: Transposable Elements Are a Significant Contributor to Tandem Repeats in the Human Genome
Source: Comp Funct Genomics. 2012 Jun 24;2012:947089. doi: 10.1155/2012/947089 (PMC3389668; doi:10.1155/2012/947089)
Supplement: Supplementary file 1 — Supplementary Material 1: Containing supplementary figures 1–3 and tables 1-2. Supplementary Figure 1: A schematic comparison for 17-repeat TR array involving the 226–278 bp region in a Alujo among difference species. Supplementary Figure 2: Box and Whiskers plot of maximum divergence among repeat units in TRs with ≤3 and ≥10 repeat units. Supplementary Figure 3: Genomic locations of all TE-derived TRs. Supplementary Table 1: The number of mlTRs at different repeat units for mlTR clusters. Supplementary Table 2: The distribution of direct repeat length for TE-derived TRs with identifiable direct repeats. Supplementary Material 2: Contains Supplementary table 3 which lists all 7,276 TE-derived TRs in a Microsoft Excel sheet. [file 947089.f1.pdf]

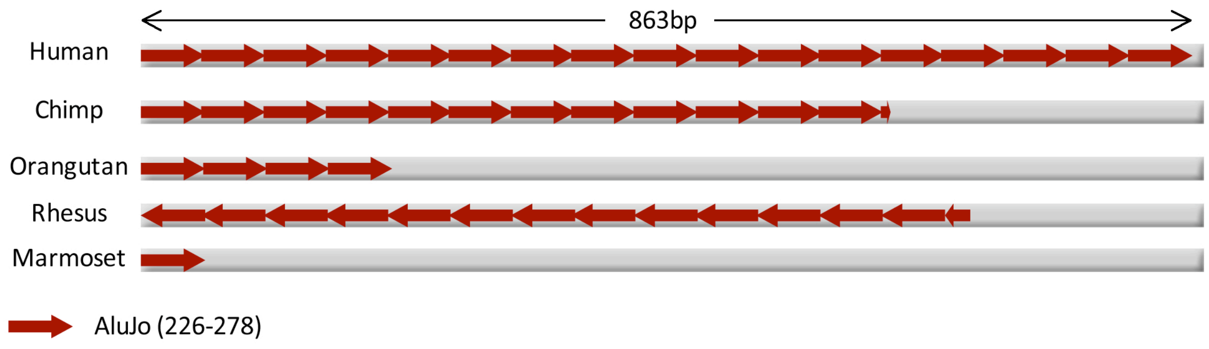

Supplementary Figure 1: A schematic comparison for a 17-repeat TR array involving the 226-278bp region in a AluJo among difference species. The human genomic region was compared with the corresponding region from chimp, orangutan, rhesus and marmoset.

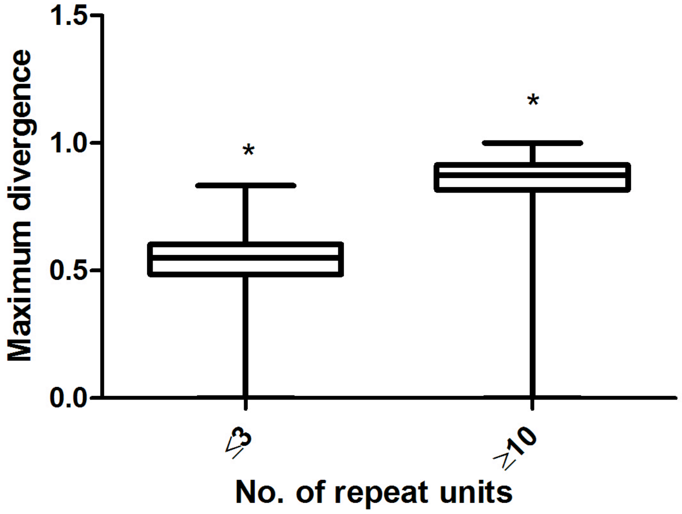

Supplementary Figure 2: Box and Whiskers plot of maximum divergence among repeat units in TRs with ≤3 and ≥10 repeat units. The mean maximal divergence among repeat units in TRs with ≤3 units is 0.5330 and is 0.8049 in TRs with ≥10 units based on all 5,902 non-LTR12 and non-L1PA TE-derived TRs. The asterisk denotes that they are significantly different (P<0.0001) in a two-tailed t-test.

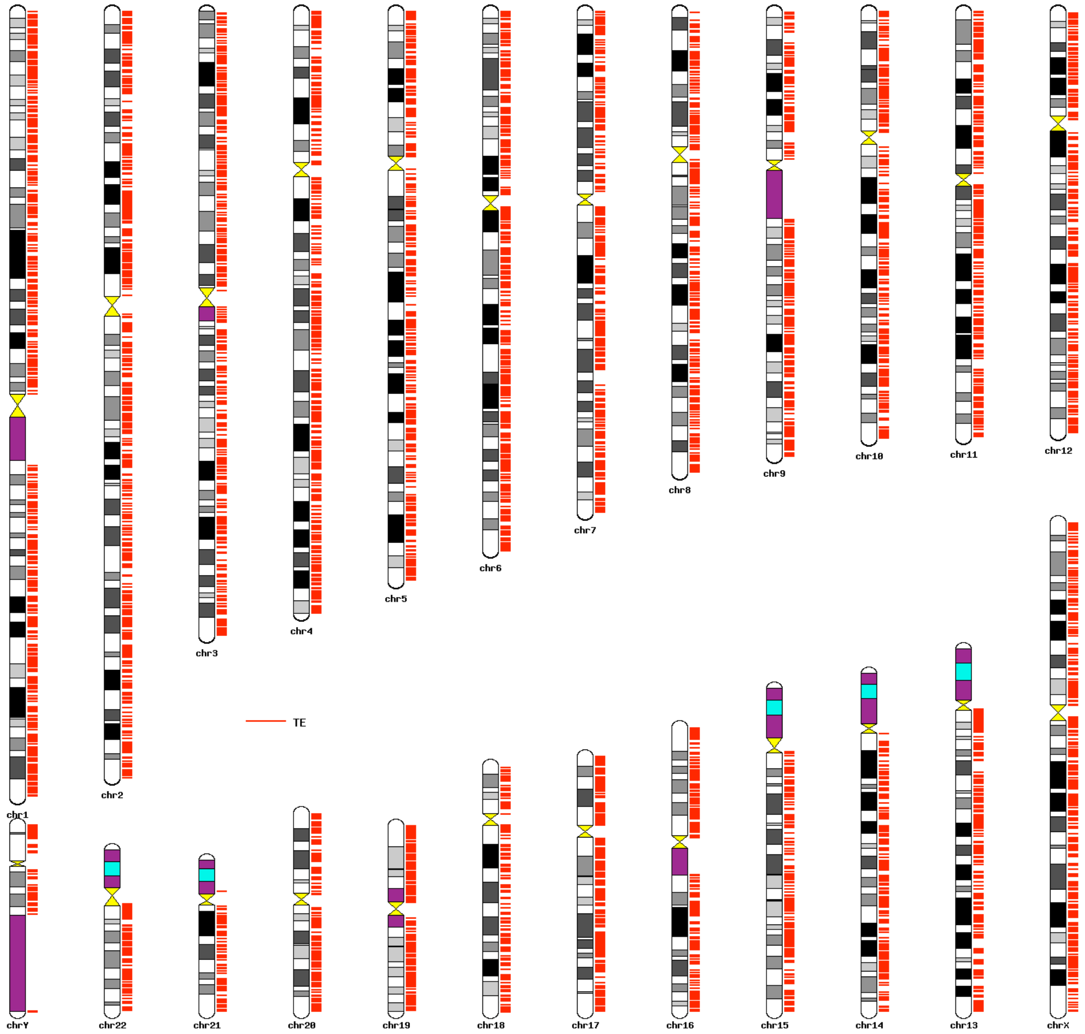

Supplementary Figure 3: Genomic locations of all TE-derived TRs. All individual TRs are plotted onto the human chromosome ideogram based on their genomic positions in the UCSC hg19 reference sequence. Chromosomal regions in color are heterochromatin regions which mostly lack sequence. Despite the ubiquitous but non-homogeneous distribution of TE-derived TRs in the genome, there seem to have no obvious hot spot.

Supplementary Table 1: The number of mlTRs at different repeat units for mlTR clusters

Cluster No.	Unit size	No. of repeat unit of each TR in the cluster	No. of different loci the TR appear in	TE subfamiliy
6	109	2	36	AluS
		3	6	
		4	1	
		17	1	
7	125	2	36	AluS
		3	5	
		5	3	
20	22	2	22	L1P3/L1PR
		3	1	
14	268	2	25	AluY
		3	3	
22	32	2	13	AluS
		3	1	
		7	1	
18	161	2	21	AluY
		3	1	
		14	2	
24	54	2	11	THE1C-int
		3	1	
32	68	2	8	HERVH-int
		3	1	
71	352	3	2	AluSx
		4	2	
55	42	2	4	HERVH-int
		3	1	
		6	1	
84	49	4	3	MER57A-int
		17	1	
259	42	2	1	HERVH-int
		4	1	
89	28	2	3	LTR10F
		8	1	
31	113	2	8	Harlequin-int
		3	1	
208	71	2	1	AluSx
		3	1	
62	42	2	3	HERVH-int
		3	1	
		4	1	

67	31	2	4	AluY/AluS
		3	1	
198	78	2	1	AluS
		3	1	
139	664	2	1	HERVE-int
		3	1	
129	24	2	2	HERVH-int
		3	1	
236	45	2	1	AluSx
		3	1	
145	255	2	1	MSR1
		4	1	
178	102	2	1	AluS
		3	1	
124	28	2	2	LTR10F
		12	1	
47	114	2	4	HERVH-int
		3	1	
		5	1	
43	25	5	1	L1M5
		6	1	
244	44	2	1	AluSq
		3	1	
74	221	2	3	AluY
		3	1	
113	39	2	1	AluY/AluS
		3	1	
39	64	2	1	HERVH-int
		4	2	
		5	1	
		6	1	
		7	2	
120	32	2	2	AluS
		3	1	
239	49	3	1	L2c
		5	1	
301	32	13	1	LTR7B
		14	1	
27	32	2	10	AluS
		3	1	
60	63	2	4	HERVH-int
		3	1	
247	46	2	1	AluSx
		4	1	
82	49	2	3	AluS
		3	1	
212	70	2	1	AluJr
		3	1	
33	61	2	5	HERVH-int

		3	4	
63	37	2	3	HERVH-int
		3	1	
		6	1	
80	93	2	3	HERVH-int
		3	1	
54	42	2	2	HERVH-int
		3	3	
		8	1	
40	60	2	5	HERVH-int
		3	1	
		9	1	
278	36	6	1	MSR1
		29	1	
34	30	2	7	AluS
		3	2	

Supplementary Table 2: The distribution of direct repeat length for TE-derived TRs with identifiable direct repeats

Direct repeat length (bp)	Number of occurrence
<7	0
7	145
8	47
9	21
10	11
11	12
12	15
13	12
14	9
15-20	28
